# Case reports: orthotic treatment of adult scoliosis patients with chronic back pain

**DOI:** 10.1186/1748-7161-9-18

**Published:** 2014-11-18

**Authors:** Dino Gallo

**Affiliations:** Ortholutions GmbH & Co. KG, Ing.-Anton-Kathrein-Straße 2, 83101 Rohrdorf - Thansau, Germany

**Keywords:** Scoliosis, Spine deformity, Customized trunk orthoses, Body, Alignment, Chronic back pain, Sagittal line

## Abstract

Orthotic treatment of patients with degenerative deformations of the spine is a complex endeavor. It is a great orthopedic technical challenge to effectively reduce the accompanying pain and to help patients regain and keep their mobility. Due to difficult therapies and poor compliance, a surgical intervention to brace the spine is usually the first therapeutic choice.

This article presents two cases in which individualized torso orthoses were successfully used to treat patients with degenerative diseases and disorders of the sagittal line as well as three dimensional deformities of the spine. Using torso orthoses allows treatment of these patients with as few invasive measures as possible without losing maximal functionality.

## Introduction

Treatment of patients with degenerative deformations of the spine and of adult scoliosis patients with chronic back pain is challenging. Accompanying orthotic services, which are mandatory in many cases, are complicated and compliance is often poor. Consequently, a surgical intervention to brace the spine is the first therapeutic choice in many cases.

Therapy of adult scoliosis patients with degenerative deformations of the spine and the accompanying chronic pains will usually improve their quality of life considerably. A stationary intensive rehabilitation is required to make these improvements permanent.

Yet in some cases the back pain and the degenerative deformations remain even after such treatments and may require a back brace. Modern orthoses are technically and biomechanically adapted to the patient and can ease the pain - or even eliminate it completely. They support and relieve the spine and contribute to an optimization of the body statics with three dimensional adapted designs. Patients use them either during specific activities or permanently [1, 2].

According to the SOSORT guidelines of 2006 and 2011, Scoliosis Intensive Rehabilitation (SIR) and orthoses are medically indicated for adult scoliosis patients with chronic pain if the therapeutic effect of the orthosis is established [3, 4]. There are several published reports that show the effects of specific physiotherapeutic treatment and the use of elastic [5] and solid braces. Some authors even describe a correction of the sagittal profile only by use of an orthosis [1, 2].

Furthermore a reduction of adult scoliosis by specific physiotherapeutic treatment is reported [6]. But it is the combination of such treatment programs such as Schroth or SEAS [6] with the use of an orthosis that has proven to be most effective [7].

One of the major tasks at hand was the development of an orthosis construction which really satisfied functional demands and would lead to a distinct improvement for the patients. To achieve this, empirical data was necessary. It was provided by monitoring more than 200 male and female adult scoliosis patients with chronic pains who have been treated with several orthotic systems for 7 years. These treatments took place as part of an intensive care program or were conducted as outpatient treatment in a specialized rehabilitation center. At first, results were mainly unsatisfactory due to immature orthosis constructions. The side effects caused by the orthosis were unacceptable compared to the improvements for the patients and rarely improved their situation significantly.

This resulted in the development of the sBrace orthosis construction [8] which employed the insights of a specialized rehabilitation center in Germany. In the beginning it was designed as a made-up TLSO for lumbar hyperextension. The advanced version can now be individualized and adapted to three dimensional indications. It affects the sagittal profile to achieve flexion or hyperextension or – depending on the individual design and demands – in a three dimensional way. The brace is manufactured individually using CAD. The specific biomechanical design of the orthosis is based on the indication and morphology of the patient as well as individual measurements and/or scans and clinical pictures. The optimal pain-free posture is determined beforehand as well.

This case report presents evidence for orthopedic technical options to reduce pain and improve quality of life for adult scoliosis patients.

## Material and method

To gather statistical data, patients from different international institutions were selected for a survey (including our patients in case 1 and 2). These patients documented the whole period of treatment, the longest case covering 9 years, the shortest only 1 year. Activities causing pain and the intensity of the pain were recorded using a scale of 0–10 (0 = no pain, 10 = worst pain ever experienced). The quality of life was likewise measured (0 = poor, 10 = excellent).

As the patients were treated in different institutions all around the world it was not possible to gather standardized reports of the treatment. The periods of use of the brace were decided by the patients themselves, so that they usually used it to perform specific activities or to counter acute pain. All patients were outpatients and received physiotherapeutic treatment.

## Results

The intensity of pain before using the orthosis averaged 7,88. During the period the patients received orthotic treatment this value was reduced to 2,63.

**Figure 1:**
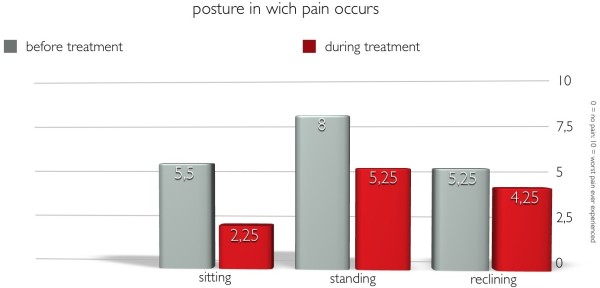
**Pain intensity of postures in which pain occurred before and during brace treatment.**

**Figure 2:**
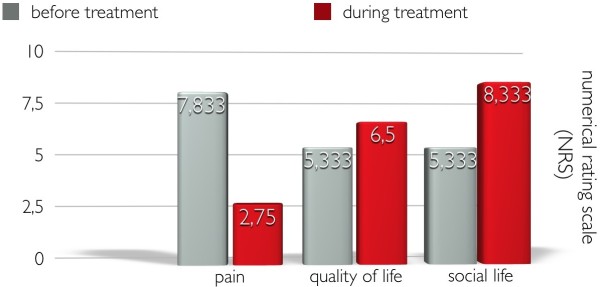
**A questionnaire with at least 13 points according to a numerical rating scale (NRS) that records the patient’s condition before, during and after the trunk orthosis treatment has been prepared for statistical evaluations.** The emphases “pain”, “quality of life” as well as “quality of social life” are depicted in the illustration. Despite the complicated fitting with trunk orthoses, an improvement could be brought about in all areas. The area of pain reduction is particularly significant.

All patient in the survey were unable to perform business, household or leisure activities or could only perform them with restrictions. When using the brace the performance of all these activities was made possible or at least improved.The intensity of pain when sitting, standing or lying could be reduced significantly by the use of the orthosis: from an average of 5,50 to 2,25 when sitting, from 8,00 to 5,25 when standing and from 5,25 to 4,25 when lying (Figure 1).Side effects of the orthosis like pressure marks were reported by 25 percent of the patients, which must be considered normal according to the circumstances. Putting the brace on and off was perceived as minor inconvenience by 25 percent. Three quarters of the patients reported no pressure marks or pains caused by the orthosis whatsoever (Figure 2).

In three cases a surgical intervention could be avoided by the use of the brace.

### Two case examples

#### Case 1: symptoms and indication

**Figure 3:**
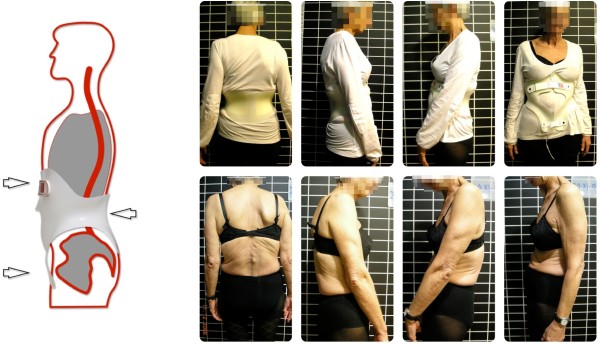
**Patient with and without the brace.** The sagittal profile of the patient is significantly closer to physiological standards.

The patient in this case was a 65 year old woman who had a spinal fusion operation. Her body did not tolerate the implants and they had to be removed. Following the operations and recuperation the original situation was restored: The diagnosis was degenerative scoliosis concomitant with kyphosis of the lumbar vertebral column and pseudarthrosis between L5 and S1.The patient (Figure 3) had multiple degenerative deformations and was suffering with severe back pain (NRS 8–9). Even minor everyday activities were impossible to accomplish: She stated that the pain prevents her from walking upright and driving even short distances. When specific physiotherapeutic treatment brought no relief, the patient chose the surgical intervention which was unsuccessful for the time being.

The independent handling of daily routines was impossible without stabilizing the lumbar spine and easing the pain. In this phase the patient assessed her quality of life and her social life as 3 on a scale of 1 to 10.

#### Case 1: Required features and design of the brace

**Figure 4:**
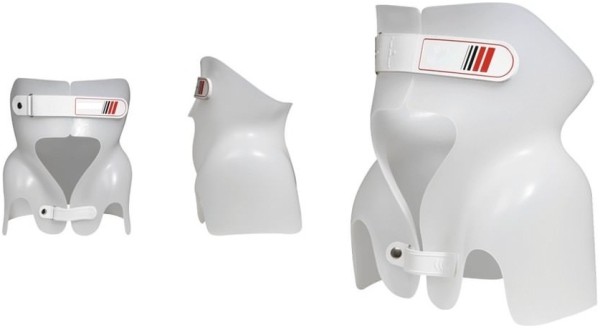
**Basic form of the sBrace trunk orthosis.** The biomechanical function can be adapted to individual requirements by customizing the layout.

**Figure 5:**
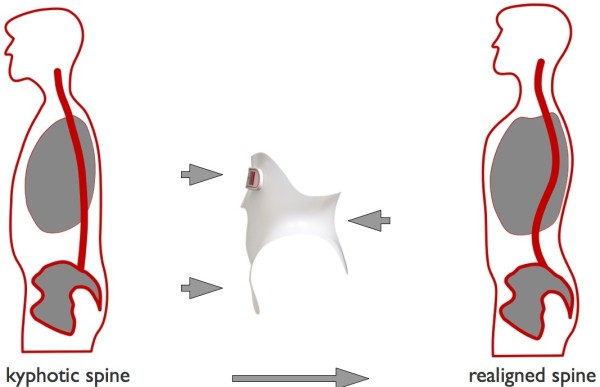
**The biomechanical function of the sBrace L with a design solely aiming to influence the sagittal profile of the spine.**

In this case the back brace had to be able to stabilize the spine in the affected lumbar segment in order to stop and reduce the stenosis of the spinal canal. This could only be achieved by changing the body statics. At the same time it had to keep the body in a pain-free position. Furthermore a correction of statics and a normalization of the sagittal profile was targeted. The functionality of the thoracolumbosacral orthosis (TLSO) had to be ensured in standing, lying and sitting position without any restriction of mobility.The patient was equipped with a sBrace L TLSO trunk orthosis module (Figures 4 and 5). The biomechanic design of the orthosis module was adapted to the necessities of the specific case during the process of ordering. It was manufactured individually according to the measurements of the patient. She did not have to endure the process of making a plaster cast.

In the next step the brace was fitted to the patient according to the indication. Thus brim courses were defined and pressure zones cushioned. By this customization the trunk orthosis module was made into a lightweight rigid TLSO in frame construction. The flexibility of the frontal area was defined by the layout of the orthesis. In this case the mobility remained completely unrestricted.

#### Case 1: results

Judging from the comparison of clinical photography the sagittal profile of the patient was improved considerably (as shown in Figure 3), which also reduced pain significantly (NRS 3–4). The patient was able to use the orthosis for specific activities in everyday life and analgesic therapy – despite the strong malposition and deformations of the spine and the resulting pressure by the TLSO. She stated that she could live an almost normal life with the brace and assessed her social life now as 8 on a scale of 1 to 10.

After a subsequent successful operation she was able to give up the orthosis.

#### Case 2: symptoms and indication

**Figure 6:**
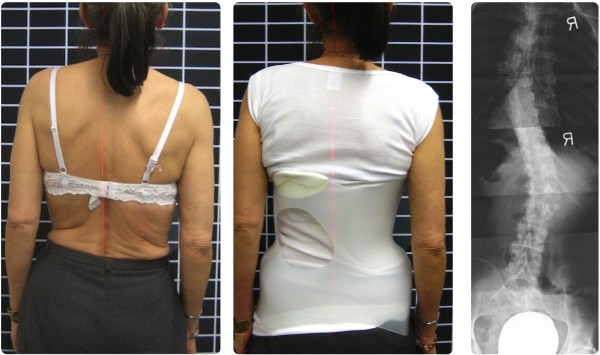
**Dorsal view.** The individualized sBrace was manufactured with both halves of the pelvis to stabilize the frontal and sagittal areas. The relocation of the pelvis to the left was necessary to make a pain-free posture possible.

**Figure 7:**
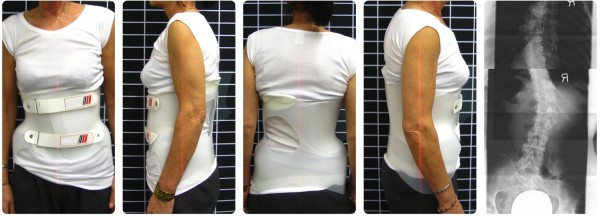
**The clinical monitoring with Lasar Posture shows a proper physiological condition of the sagittal profile.**

The second example case is a patient (Figures 6 and 7) with degenerative scoliosis and long-term consequences of vertebral body fractures between L4 and Th8 concomitant with spondylolisthesis between L3 and L4. These resulted in chronic pain (NRS 8) which made independent mobility very difficult or even impossible. The patient reported severe pain especially when walking or lying down – the latter making her sleep troublesome und thus her life even more strained.

A surgical intervention was commended to treat the spondylolisthesis but the patient rejected it.

#### Case 2: required features and design of the brace

In this case the orthosis had to be able to stabilize the lumbar and thoracolumbar segments of the spine. The objective was to change the pathomechanic statics of the vertebral column to improve the spondylolisthesis and enable a pain-free posture.

For this patient the basic form of the sBrace L orthosis module was selected. The brace was manufactured according to the measurements and clinical pictures of the patient without plaster cast. The deviation of the pelvis to the right was integrated into the CAD model. The adaptation of the physiological sagittal profile of the brace’s basic form was based on clinical tests and the patient’s morphology. Furthermore, the left thoracic, the right lumbar and the left gluteal layout were integrated into the CAD model to ensure the three point pressure system to stabilize the frontal area. In the CAD model the pressure points were adapted to the morphology of the patient and to functional requirements.

Thus the biomechanically standardized basic form of the brace was individually manufactured by cutting the brim according to the requirements of the individual patient. Sensitive pressure zones were cushioned.

#### Case 2: results

The progression of the spondylolisthesis was stopped by the use of the brace and continued to be stable, as shown in Figures 6 and 7. According to the attending physician surgical intervention was avoided. The pain was reduced considerably (NRS 2) and the patient’s mobility in everyday life was ensured. Even leisure activities like light exercise were made possible by the orthosis.

## Discussion

The success of orthotic treatment depends strongly on the experience that all involved professions have gathered with the patients in question. The manufacturing and even more the adaptation of an orthosis must be conducted with the utmost accuracy and patience. The CPO has to take his time to adapt the brace step by step together with the patient.

To get positive effects the following points have to be observed:

1. thorough evaluation and clinical tests
2. extensive education of the patient and definition of the purpose of the treatment
3. profound knowledge of the orthosis design
4. experience in adapting the orthosis construction
5. experience and empathy in dealing with patients

The education of the patient before the start of treatment is of special importance. Particularly the side effects must be made clear. The patient must learn that it may take some time until the use of the brace becomes a habit. Usually even when it is put on for the first time the patient feels some relief. But especially at the beginning regular meetings to adapt the brace will be necessary. With these adaptations it is possible to implement learnings which only the everyday use of the orthosis by the specific patient can bring.

## Conclusion

Orthotic treatment of patients with degenerative deformations of the spine is a complex endeavor. It is a great orthopedic technical challenge to effectively reduce or eliminate the accompanying pain and to help patients regain and keep their mobility to manage their everyday activities.

The evaluation of the statistical data show that individualized torso orthoses can successfully be used for analgesic treatment of patients with degenerative diseases and disorders of the sagittal profile. The example cases demonstrate that these options can be used in acute as well as in temporary or permanent treatment for outpatient care. And although all covered cases relate to outpatients, the data suggests that inpatients would benefit from the treatment as well.

Using torso orthoses allows treatment of these patients with as little invasive measures as possible without losing maximal functionality. Profound knowledge and a systematic care in planning are mandatory to implement this kind of orthotic treatment.

Interdisciplinary cooperation of all professions involved in the treatment and an accompanying specific physiotherapeutic treatment is obligatory for achieving the best possible result for the patient.

### Consent

Written informed consent was obtained from the patient for the publication of this report and any accompanying images.

## Authors’ information

Dino Gallo is co-founder and CEO of Ortholutions GmbH & Co. KG in Germany. He is German certified prosthetist and orthotist, author of the RSC® (Rigo System Chêneau) brace, and a founding member of the SOSORT.
